# A Power-Aware 5G Network Slicing Scheme for IIoT Systems with Age Tolerance

**DOI:** 10.3390/s25226956

**Published:** 2025-11-14

**Authors:** Mingjiang Weng, Yixuan Bai, Xin Xie

**Affiliations:** 1 School of Communications and Information Engineering, Chongqing University of Posts and Telecommunications, Chongqing 400065, China; wengmj@cqupt.edu.cn (M.W.); d240101001@stu.cqupt.edu.cn (Y.B.); 2 Key Laboratory of Industrial Internet of Things and Networked Control, Chongqing University of Posts and Telecommunications, Chongqing 400065, China

**Keywords:** network slicing, 5G, Age of Information, scheduling

## Abstract

Network slicing has emerged as a pivotal technology in addressing the diverse customization requirements of the Industrial Internet of Things (IIoT) within 5G networks, enabling the deployment of multiple logical networks over shared infrastructure. Efficient resource management in this context is essential to ensure energy efficiency and meet the stringent real-time demands of IIoT applications. This study focuses on the scheduling problem of minimizing average transmission power while maintaining Age of Information (AoI) tolerance constraints within 5G wireless network slicing. To tackle this challenge, an improved Dueling Double Deep Q-Network (D3QN) is leveraged to devise intelligent slicing schemes that dynamically allocate resources, ensuring optimal performance in time-varying wireless environments. The proposed improved D3QN approach introduces a novel heuristic-based exploration strategy that restricts action choices to the most effective options, significantly; reducing ineffective learning steps. The simulation results show that the method not only speeds up convergence considerably but also achieves lower transmit power while preserving strict AoI reliability constraints and slice isolation.

## 1. Introduction

The rapid advancement and widespread deployment of 5G cellular networks have significantly facilitated the adoption of Industrial Internet of Things (IIoT) applications, including remote factory management systems, intelligent industrial equipment, and real-time monitoring. These applications leverage a massive number of interconnected intelligent devices, each demonstrating diverse Quality of Service (QoS) requirements and highly variable traffic characteristics. To address these challenges, 5G radio access network (RAN) slicing technology enables the dynamic allocation and scheduling of wireless resources, ensuring the isolation and customization necessary to meet the unique demands of IIoT applications while maintaining system efficiency and reliability [1].

Recently, RAN slicing has drawn significant attention due to its potential for efficient resource utilization and service customization in 5G networks. Several studies have proposed various solutions to optimize resource management and improve the performance of RAN slicing. In [2], the authors present a two-timescale resource management scheme to maximize tenant profit by dynamically adjusting resource allocation. In [3], a spectrum resource optimization algorithm is developed, leveraging historical traffic data and an intelligent resource scheduling strategy based on traffic prediction. A Lyapunov drift-plus-penalty approach is proposed in [4] to minimize the consumption of physical resource blocks (PRBs) while ensuring slice isolation. This method dynamically assigns resources to slices, satisfying their average rate and delay requirements. The authors of [5] introduce a data-driven RAN slicing mechanism that employs a resource-sharing algorithm at the Slice Orchestrator (SO) level. This approach meets the stringent requirements of two major slice types in 5G networks. Furthermore, in [6], a two-level soft-slicing scheme is designed to satisfy the strict quality of service requirements for ultra-reliable low-latency communication (URLLC) and enhanced mobile broadband (eMBB) services while minimizing the total number of PRBs used at the network level.

While the aforementioned works primarily focus on resource optimization for RAN slicing, they often underemphasize energy consumption considerations. Infrastructure providers, however, face significant challenges related to both data transmission and energy efficiency. Reducing network energy consumption depends not only on overall infrastructure deployment but also on the energy performance of individual Base Stations (BSs). Notably, the radio frequency (RF) module contributes the largest share to a BS’s energy consumption [7,8]. In [9], the authors propose a power consumption minimization strategy based on the Lyapunov drift-plus-penalty method, which ensures slice isolation and Quality of Service (QoS). The study in [10] introduces a deep reinforcement learning (DRL) framework that minimizes average power consumption while achieving ultra-reliable low-latency communication through model-free resource allocation. Unlike traditional algorithms, DRL approaches require no strict system modeling or assumptions, enabling direct learning and optimization in real-world environments. This makes DRL methods particularly well-suited to handling the complexity and uncertainty inherent in wireless environments. As highlighted in [11] AI-/ML-supported techniques hold the potential to enable future networks to adapt intelligently to uncertain and dynamic environments, significantly improving performance metrics such as reliability and latency. To address the challenges posed by the large state space and partial observability in such systems, distributed algorithms offer a promising avenue for mitigating computational costs. Moreover, next-generation wireless networks can aggregate substantial computational resources and data from a vast array of wireless devices, as discussed in [12]. This capability provides a potential solution to challenges associated with computational demands and energy supply in artificial intelligence-driven networks. Moreover, some works adopt modified DRL approaches according to the application scenarios, including enhanced DDPG [13] and TD3 algorithm [14], demonstrating significant performance improvements.

Apart from minimizing energy consumption, another challenge is how to meet the real-time requirements for IIoT applications [15,16]. AoI is first introduced by Kaul et al. as an emerging real-time performance metric that quantifies the freshness of information in communication systems by jointly capturing data transmission delays and the rate of information generation [17]. It is defined as the time elapsed since the most recently generated data packet is successfully received. AoI is widely recognized as an effective indicator for evaluating the timeliness and reliability of information delivery in time-critical applications and has found widespread applications in various communication and networking scenarios [18,19]. For example, in an IIoT system that rely on autonomous intelligent control decisions, the information transmitted by data becomes outdated over time, and the destination cannot receive reliable data on time, resulting in critical production accidents. Therefore, it is essential to ensure that the received data meets specific freshness threshold constraints. The AoI tolerance threshold specifies the maximum acceptable delay for critical data. Industrial applications require strict AoI tolerances such as real-time control with less than 10 ms, condition monitoring between 100 ms and 1 s, and non-critical monitoring exceeding 1 s. We investigate the scheduling problem of minimizing average downlink transmit power in an IIoT system under the constraint that different RAN slices meet the corresponding AoI tolerance. The main contributions are summarized as follows:

- We consider using 5G RAN slice to ensure the reliability of AoI and reduce energy consumption in the case of time-varying channels and time-varying traffic in the IIoT system scheduling.
- An allocation algorithm based on Dueling Double Deep Q-Network is proposed to minimize the consumption of transmitting power while ensuring AoI tolerance and slice isolation in 5G-enabled IIoT systems.
- We have designed a novel heuristic-based exploration algorithm that accelerates the convergence time and improves the steady-state performance of D3QN. Simulation results verify the effectiveness of the proposed algorithm.

The rest of this paper is structured as follows. In Section 2, system model and optimization problem are presented. We propose the dynamic PRBs allocation method based on deep reinforcement learning in Section 3. Section 4 provides the simulation results of the proposed algorithm. The conclusion is drawn in Section 5.

## 2. System Model and Problem Formulation

In this section, we consider a 5G IIoT system with a single BS downlink including *N* IIoT nodes and *M* network slices, where $i\in \left\{1,\ldots ,N\right\}$ and $n\in \left\{1,\ldots ,M\right\}$ represent the index of nodes and network slices respectively. The BS maps *N* nodes to *M* slices according to the QoS requirements of the nodes through virtualization system physical resources [4]. By adjusting the communication bandwidth online, the QoS of each node is guaranteed, and the isolation between slices is realized. The downlink communication bandwidth is divided into *K* PRBs, and each PRB is set to include *B* MHz, where the index of PRB is represented by $j\in \left\{1,\ldots ,K\right\}$. The *M* slices are set to have different maximum tolerable AoI $A_{n}^{max}$ and target reliability $\Omega_{n}^{max}$ quality of service requirements. If the AoI of the data is greater than the threshold $A_{n}^{max}$, it indicates that the data has expired and has no use value, while the reliability is used to ensure that the amount of timeout data is small enough that it does not affect the normal operation of the system.

**Figure 1:**
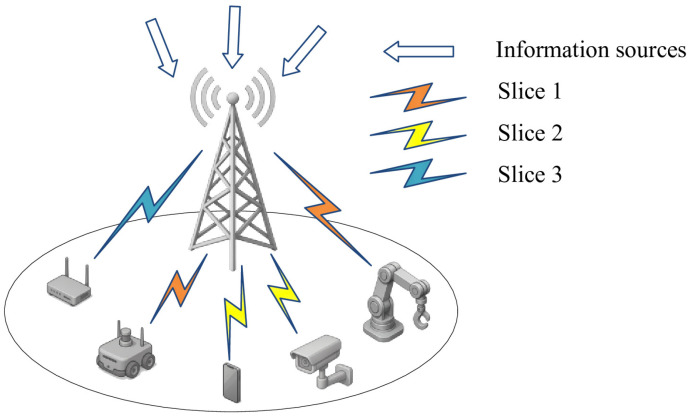
The scenario diagram.

The system is based on time slot. In this system as shown in Figure 1, we need to dynamically allocate $k_{i}$ PRBs to node *i* in each time slot *t*, where satisfies the constraint condition $K=\sum_{i\in N}k_{i}$. In addition, the channel between the BS and the node is set to change independently between time slots but remains unchanged in the time slots. Specifically, the channel gain $h_{i,j}^{t}$ between the BS and the node *i* on the PRB *j* is assumed to conform to the time-varying Rayleigh channel and Log-distance path-loss model. As a consequence, according to Shannon’s formula, the transmission rate between the BS and the node can be expressed as

(1) $$r_{i}^{t}=B\sum_{j\in K}\rho_{i,j}^{t}\log_{2}\left(1+\frac{p_{i,j}^{t}\cdot h_{i,j}^{t}}{\sigma^{2}}\right),$$

where $p_{i,j}^{t}$ is the transmission power of the BS on PRB *j* in slot *t*, and $\sigma^{2}$ is the thermal noise on each PRB. The $\rho_{i,j}^{t}$ is an indicator function, which means that, if PRB *j* is allocated to node *i* at time slot *t*, $\rho_{i,j}^{t}=1$, else, $\rho_{i,j}^{t}=0$.

Besides, we define two variables $A_{i}^{s}$ and $A_{i}^{d}$ as AoI at the BS and AoI at the node, respectively. In every time slot, the AoI grows linearly in the absence of new packets generated at the BS or data being delivered to the node. Once the node successfully receives data from the BS, its AoI is updated to match the current AoI of the BS. In this paper, we assume that the data generated at the BS for different users are independent of each other and are also independent and identically distributed (i.i.d.) over time slots, following a Bernoulli distribution. Once new data is generated, $A_{i}^{s}$ is set to 0. The evolution of $A_{i}^{s}$ and $A_{i}^{d}$ is as follows:

(2) $$A_{i}^{s}\left(t+1\right)=\left\{\begin{array}{cc} 0, & \;\mathrm{if}\;\mathrm{new}\;\mathrm{data}\;\mathrm{is}\;\mathrm{generated},\\ A_{i}^{s}\left(t\right)+1, & \;\mathrm{otherwise}. \end{array}\right.$$

(3) $$A_{i}^{d}\left(t+1\right)=\left\{\begin{array}{cc} A_{i}^{s}\left(t\right)+1, & \;\mathrm{if}\;\mathrm{the}\;\mathrm{data}\;\mathrm{is}\;\mathrm{delivered},\\ A_{i}^{d}\left(t\right)+1, & \;\mathrm{otherwise}. \end{array}\right.$$

Given this model, the optimization objective in (4) aims to formulate a scheduling policy that minimizes average transmission power while ensuring AoI constraints and slice isolation within different 5G wireless network slicing. This problem is a mixed-integer stochastic optimization which is an NP-hard problem.

(4) $$\begin{array}{cc} \; & min\lim_{T\to \infty }\frac{1}{T}\sum_{t=0}^{T-1}\sum_{i=1}^{N}p_{i}\left(t\right),\\ \; & s.t.\;C1:\;\sum_{i\in N}\rho_{i,j}=1,\\ \; & \;C2:\;0\leq \sum_{j\in K}\sum_{i\in N}\rho_{i,j}\leq K,\\ \; & \;C3:\;{0\leq p}_{i}\leq p_{max},\\ \; & \;C4:\;P\{A_{n}^{d}<A_{n}^{max}\}\geq \Omega_{n}^{\max}. \end{array}$$

The constraints in C1, C2, and C3 mean that the slices are isolated from each other, the total number of PRBs allocated is less than the number of PRBs, the power is less than the maximum power $p_{max}$. Constraint C4 guarantees the reliability of AoI, which can be regarded as the probability of AoI not expired. In practical wireless communication systems, the unavailability of precise channel and data traffic models frequently prevents the derivation of exact analytical solutions. However, reinforcement learning methods enable optimization strategies through the interaction between environment and agent, thereby enhancing applicability to practical scenarios.

## 3. Scheduling Algorithm

In this section, a scheduling algorithm is proposed based on deep reinforcement learning, which minimizes the transmit power usage of the BS, through the allocation of PRBs, under the premise of satisfying the AoI constraints.

The optimization of the objective formula can be regarded as the optimal strategy choice, which is the allocation problem of physical resource blocks, and can be transformed into a dynamic scheduling problem. The classic solution to the optimal policy selection problem is to use Markov Decision Processes (MDP), but traditional MDP relies on the state and the *Q* value corresponding to each action of the state. Therefore, when the state space grows exponentially, the computational complexity of solving an optimization problem by value iteration increases dramatically that it becomes impossible to solve. Compared with MDP, deep reinforcement learning can use a neural network to realize the input state value and output the *Q* value corresponding to all action values, so as to solve the problem of large state space.

In order to better solve the scheduling problem of large action space in complex environment, this paper adopts the algorithm based on deep reinforcement learning (DRL). As a classic DRL algorithm, the deep *Q*-Network (DQN) model is widely used to solve scheduling optimization problems. It approximates the action value function through a neural network and then selects the optimal action corresponding to the state according to the *Q* value to complete the scheduling. In addition, since the states based on time slots are continuous, the neural network trained in this way is prone to overfitting. In order to solve this problem, the replay memory is adopted in the DQN, the training data is saved in the replay memory, and the data is randomly selected from the replay memory for training each time, which ensures that the training samples are independent and identically distributed.

It is worth noting that, to alleviate overestimation and increase the stability of the algorithm, DQN includes two networks, evaluation networks, and target networks, with the same structure but different parameters, in which the evaluation network uses the latest parameters, while the target value network uses the past parameters, and each several steps update the parameters of the evaluation network to the target network.

The D3QN algorithm is an enhancement of the DQN algorithm. Firstly, two branches are included in the output to improve learning efficiency. By combining the state value of the state with the advantage value of each action, the *Q* value corresponding to each action is calculated. Secondly, using different networks to select and evaluate actions for mitigating overestimation in action value estimation. Optimal action is selected according to the maximum *Q* value of the evaluation network at time slot t+1 and then uses the $Q^{\prime }$ value corresponding to the action in the target network to calculate the $Q_{Ta}$ value at time *t*, i.e.,

(5) $$Q_{Ta}=R_{i,t}+\gamma Q^{\prime }\left(s_{t+1},\underset{a_{t+1}}{\arg\max}Q\left(s_{t+1},a_{t+1};\theta \right);\theta^{\prime }\right),$$

where $s_{t}$, $a_{t}$, *R*, γ, θ, and $\theta^{\prime }$ is the system state, the action, the reward, the attenuation factor, the evaluation network parameters and the target network parameters, respectively.

Specifically, in this paper, the system state at time slot *t* is defined as the set of the AoI of the source $A_{t}^{s}$, the AoI of the destination $A_{t}^{d}$, the admitted traffic α, and the channel gain $h_{t}$ of each node, so the system state $s_{t}$ at time slot *t* can be given by

(6) $$s_{t}=\left\{A_{t}^{s},A_{t}^{d},\alpha ,h_{t}\right\},$$

where $A_{t}^{s}={\left\{A_{i,t}^{s}\right\}}_{i\in N}$, $A_{t}^{d}={\left\{A_{i,t}^{d}\right\}}_{i\in N}$, $\alpha ={\left\{\alpha_{i}\right\}}_{i\in N}$ and $h_{t}={\left\{h_{i,t}\right\}}_{i\in N}$, respectively. In (6), α is the slice rate guarantee provided by the infrastructure provider. In a real-world system, these state parameters can be obtained by the agent through the measurement and control channels of the BS and computed at the BS side.

Generally, the number of PRBs is much smaller than the division granularity of power. Therefore, the complexity of using the power distribution method as the action space is much higher than that of using the PRBs distribution method as the action space. Furthermore, in the case of known traffic rate, PRBs numbers along with channel quality, the required power can be calculated by (1), so the action space in this paper is defined as a set of all possible allocations of PRBs.

The action space *a* can be expressed as

(7) $$a={\left\{a_{i}\right\}}_{i\in N},$$

where $a_{i}$ represents the allocation set of each node *i*. In every time slot, the action is chosen by employing random scheduling following the ε−greedy policy

(8) $${\vec{a}}_{t}=\left\{\begin{array}{cc} \arg\max_{a_{t}}Q\left(s_{t},a_{t};\omega \right), & \mathrm{with}\;\mathrm{probability}\;1-\varepsilon ,\\ \mathrm{randomly}\;\mathrm{chosen}\;\mathrm{from}\;a, & \mathrm{with}\;\mathrm{probability}\;\varepsilon . \end{array}\right.$$

In (8), ε represents exploration rate decreasing with convergence of the algorithm, and the *Q* function describing the dueling network with parameter ω comprises both the state value function and the advantage function [20]. The exploration rate decreases gradually during the convergence of the DRL algorithm. Specifically, the decay rate of the exploration rate needs iterative adjusting based on the actual application scenario to optimize performance.

In this paper, considering the optimization objective in (4), we reduce the action space for ineffective exploration in (8). The new exploration space *e* is as follows:

(9) $$\begin{array}{cc} e= & \{e_{i}\in a|e_{i}\;is\;one\;of\;the\;l\;elements\;with\;\\ & the\;largest\;\sum_{i\in N}\sum_{j\in K}B\cdot \rho_{i,j}^{t}\cdot \frac{h_{i,j}^{t}}{\sigma^{2}}\} \end{array}$$

In wireless communication, both increased bandwidth and higher signal-to-noise ratio lead to reduced transmit power according to (1). In (9), we use the product of both as the selection criterion for exploring the action space and select the top *l* values as the new action space *e*. Thus, the random selection action space in the exploration process of (8) changes from *a* to *e*. We apply this strategy to the D3QN algorithm, referring to it as the improved D3QN.

The reward function is the feedback of the selected action ${\vec{a}}_{t}$ in state $s_{t}$, which is used to evaluate the outcome of the selected action. Penalties need to be generated when the constraints are not met, otherwise, rewards are given, and the more in line with the optimization goal, the greater the reward. Therefore, the reward function of any node *i* at time slot *t* is defined as

(10) $$R_{i,t}\left\{s_{t},{\vec{a}}_{t}\right\}=\left\{\begin{array}{cc} p_{max}-p_{i,t}, & \Omega_{i,t}\leq \Omega_{n}^{\max}\\ p_{max}-p_{i,t}-\\ \;\Theta \left(K_{i,t}-K_{i,t-1}\right), & \Omega_{i,t}>\Omega_{n}^{\max}, \end{array}\right.$$

where $R_{i,t}$, $p_{i,t}$ and $K_{i,t}$ is the reward, the transmission power and the proportion of the AoI at the source that exceeds the threshold of node *i* at time slot *t* respectively, and Θ is the weight, used to adjust the size of the penalty.

Furthermore, the reward function $R_{t}$ directly influences neural network updates and can be obtained as the sum of all node rewards

(11) $$R_{t}=\sum_{i\in N}R_{i,t}\left\{s_{t},{\vec{a}}_{t}\right\},$$

The Equation (11) computes overall system performance by adding up individual node rewards and transforms complex resource allocation challenges to quantifiable optimization targets.

**Figure 2:**
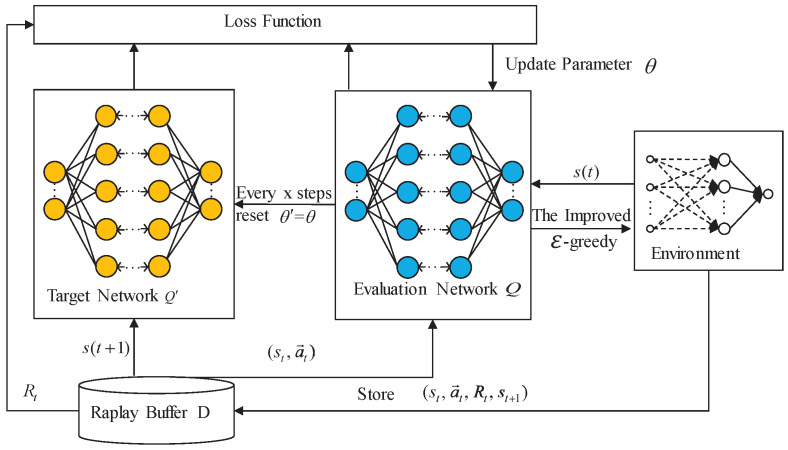
The improved D3QN algorithm framework.

In summary, the workflow of the algorithm proposed in this paper is described in Algorithm 1. The framework of improved D3QN is shown in Figure 2.
**Algorithm 1** Training process of the improved D3QN1:Input: Total iteration rounds *T*, attenuation factor γ, and exploration rate ε.2:Initialize the evaluation network parameters θ, target network parameters $\theta^{\prime }=\theta$, $\theta^{\prime }$ update frequency *x*, and replay buffer *D*;3:**for** t=1 to *T* **do**4:      Randomly select actions according to the probability of ε in the optimized exploration space *e*, otherwise select actions according to the greedy policy;5:      Taking action, the agent gets new status $s_{t+1}$ and reward;6:      Store combination $\left(s_{t},{\vec{a}}_{t},R_{t},s_{t+1}\right)$ to *D*;7:      Randomly select a batch of $\left(s_{t},{\vec{a}}_{t},R_{t},s_{t+1}\right)$ from *D* and calculate *Q*-Value of the evaluation network;8:      Calculate loss and update parameters θ;9:      Every *x* steps reset $\theta^{\prime }=\theta$, update the target *Q*-values, and reduce ε value;10:     $s_{t}=s_{t+1}$;11:**end for**

In general, the complexity of the improved D3QN algorithm primarily depends on the neural network training process and the action selection mechanism. Let $N_{l}$ denote the number of neurons in each layer, $L_{n}$ the number of layers in the neural network, and $T_{t}$ the number of training iterations. The computational complexity of a single forward pass through the network is $O(N_{l}^{2}L_{n})$, and the overall training complexity can be approximated as $O(T_{t}N_{l}^{2}L_{n})$. In practical applications, Once the model is trained, the action selection process is computationally lightweight and the agent employs the learned policy to perform resource scheduling based on the current state. Therefore, the stringent real-time scheduling requirements can be satisfied in wireless communication systems.

## 4. Results

This section will simulate the algorithm proposed in this paper and analyze the simulation results. Specifically, the radius of the BS is set to 2 km [9], and the bandwidth of each PRB is set to 1 MHz. The maximum transmit power of BS is assumed to be 46 dBm, and the noise power spectral density is −174 dBm/Hz [6]. Rayleigh fading channel is used for the links between the BS and nodes experience independent and identically distributed. In addition, the carrier frequency is configured at 1.8 GHz. The BS contains two slices, and each slice contains one node. The initial location of the node is randomly set to any location within the cell. The timeout threshold for the constraint AoI of the slice is set to 10. Moreover, the reliability of the two slices are set to 0.99 and 0.95, and the number of PRBs is set to 6, respectively. To ensure the reliability of the simulation, each experiment was conducted for 10 runs.

**Figure 3:**
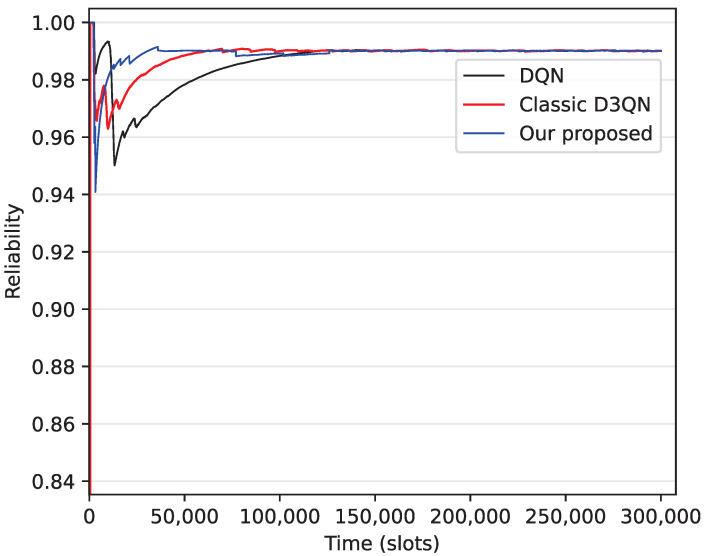
Convergence of the algorithm.

In order to evaluate convergence of the algorithm, in Figure 3, we show the process by which AoI reliability reaches the target value 0.99. From this figure, we can see that the convergence times for the DQN, D3QN, and the improved D3QN algorithms are approximately 150,000 time slots, 100,000 time slots, and 45,000 time slots, respectively. Furthermore, compared to DQN, D3QN accelerates convergence speed by achieving a more stable and efficient learning process through more accurate state value estimation and by addressing overestimation bias, while the improved D3QN optimizes the random exploration process of D3QN, reducing ineffective exploration and further enhancing convergence speed.

Furthermore, the algorithm proposed in this paper has been compared with the proportion fair (PF) algorithm [21]. The PF algorithm is a heuristic approach that allocates PRBs based on traffic proportion. In the PF algorithm, the ratio of the number of PRBs among different nodes is the same as that of the traffic between nodes.

**Figure 4:**
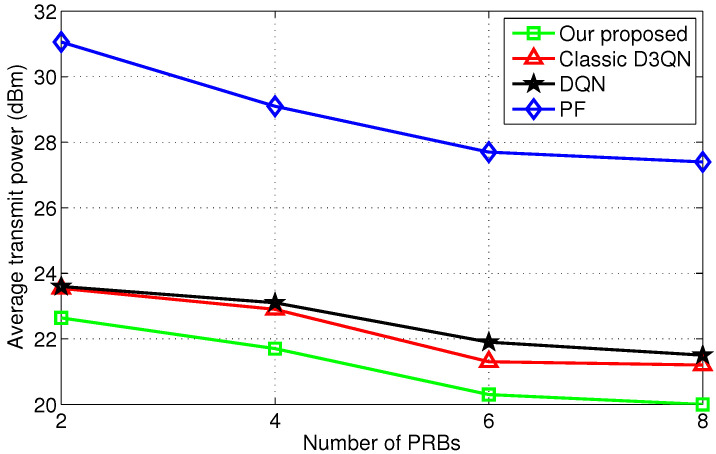
Comparison of scheduling under different number of PRBs.

Figure 4 illustrates that the average transmit power decreases with the number of PRBs, as expected, since increasing the number of PRBs allows the system to reduce average transmission power through more refined power control while ensuring transmission quality. Benefiting from continuous improvements over D3QN and DQN, the improved D3QN achieves further performance enhancements among the evaluated algorithms. Unlike reinforcement learning algorithms, traditional PF algorithms cannot provide optimal performance due to insufficient consideration of the variability of the wireless channel. They are typically based on predefined rules and are insensitive to dynamically changing environments.

**Figure 5:**
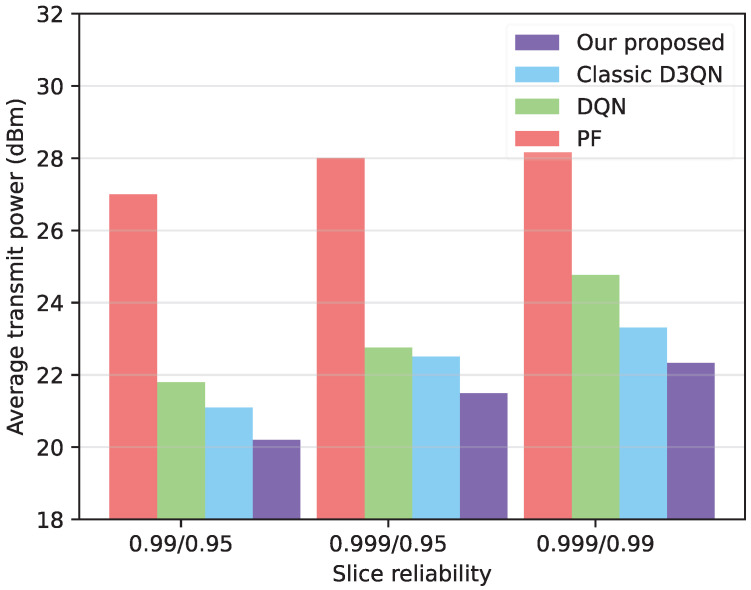
Comparison of scheduling under different reliability.

In Figure 5, the generalization of the model is verified by altering the AoI reliability. It can be observed that the improved D3QN algorithm proposed in this paper yields favorable results when different slices have different reliabilities. This results in lower transmit power compared to the PF algorithm, and outperforms the D3QN and DQN algorithm. In addition, with the improvement of user reliability requirements, the average transmission power of the BS is expected to increase. Furthermore, with the increase in user reliability requirements, the accuracy of the optimization algorithm in scheduling PRBs becomes more critical, leading to an increase in the average transmission power.

**Figure 6:**
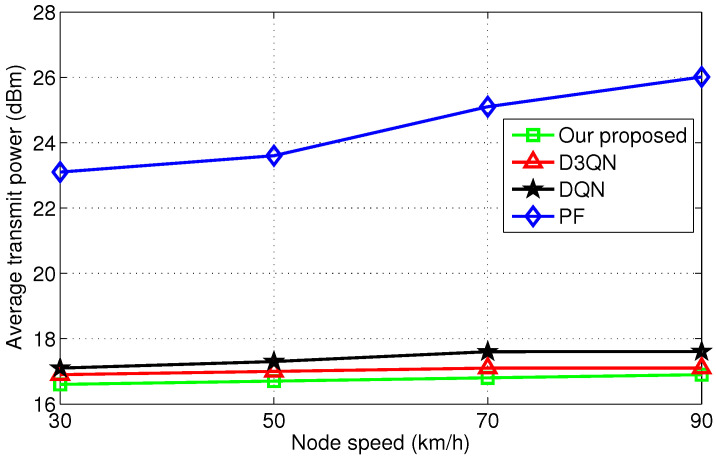
Comparision of scheduling under different speed.

As shown in Figure 6, the average transmit power of the PF algorithm increases with node speed, whereas reinforcement learning algorithms exhibit no significant increase. It is for this reason that the resource scheduling and allocation of reinforcement learning algorithms can adapt to channel dynamics in real-time. Meanwhile, the average transmit power consumption of the improved D3QN algorithm performs the best among the compared algorithms.

## 5. Discussion

The results show that the proposed method greatly improves convergence speed while meeting reliability requirements and reducing downlink RF transmission power. The improved D3QN algorithm converges faster than both DQN and standard D3QN. This improvement comes from a more efficient exploration strategy that reduces unnecessary learning steps and makes training more stable. A faster convergence rate is particularly important in IIoT systems, where real-time decision-making and fast adaptability are required to handle dynamic network conditions.

A key challenge in IIoT networks is delivering information on time while using resources efficiently. AoI is an important metric because it measures both transmission delays and how often information is updated. In time-sensitive IIoT applications, outdated information can lead to incorrect control decisions, affecting system reliability and efficiency. The proposed algorithm balances AoI requirements with energy efficiency, keeping data fresh while reducing power consumption. This balance is critical for applications such as industrial automation, remote monitoring, and predictive maintenance, where real-time data is essential for safe and efficient operations.

Unlike traditional PF algorithms, which use fixed scheduling rules and cannot adapt well to changes in the wireless environment, reinforcement learning-based methods, such as the improved D3QN, adjust resource allocation in real time based on system conditions. This adaptability allows better performance in scenarios where wireless channels and traffic loads change unpredictably. By continuously learning from network variations, the proposed method provides more stable and efficient resource allocation, improving both transmission reliability and energy efficiency.

While this study primarily focuses on the power-AoI tradeoff, the proposed algorithm’s faster convergence and intelligent resource allocation inherently contribute to improved latency performance and throughput efficiency. The reward function design also ensures balanced service provision across slices.

The results also show that the improved D3QN works well when different slices have different reliability requirements. When user reliability demands increase, the system must allocate resources more carefully, which leads to higher transmission power consumption. This tradeoff highlights the need for intelligent scheduling strategies that not only reduce power usage but also maintain slice isolation and QoS. Future work will focus on developing distributed learning approaches to handle larger state spaces efficiently. Additionally, federated learning techniques will be explored to enable collaborative optimization across multiple network nodes while keeping data privacy secure. Other potential improvements include integrating edge computing to further reduce latency and combining model-based optimization with reinforcement learning for better decision-making in complex IIoT environments.

## 6. Conclusions

In this paper, we implemented customized services for latency-sensitive nodes within a single Base Station IIoT scenario using a network slicing approach. We utilized an online PRB scheduling mechanism to minimize the average transmit power while maintaining the AoI tolerance constraints. Additionally, an improved D3QN algorithm was introduced to develop the scheduling policy. The reward function was specifically designed to ensure convergence by optimizing the exploration strategy to reduce unnecessary learning steps and maintaining higher learning stability in dynamic wireless environments.

The results demonstrate that the proposed technique accelerates convergence and effectively ensures reliability requirements while reducing downlink RF transmit power. Real-time decision-making and rapid adaptability are importent for handling dynamic network conditions in IIoT. Our proposed algorithm scheme simultaneously meets the different reliability demands and achieves an optimal balance between energy efficiency and information freshness. This feature is particularly significant for time-sensitive applications such as industrial automation and remote monitoring.

In future work, we plan to introduce distributed algorithms to address applications with exponentially expanding state spaces.

## Data Availability

Data are contained within the article.
